# West Nile virus lineage 2 in Romania, 2015–2016: co-circulation and strain replacement

**DOI:** 10.1186/s13071-018-3145-5

**Published:** 2018-10-26

**Authors:** Ani Ioana Cotar, Elena Fălcuță, Sorin Dinu, Adriana Necula, Victoria Bîrluțiu, Cornelia Svetlana Ceianu, Florian Liviu Prioteasa

**Affiliations:** 1 Cantacuzino National Medico-Military Institute for Research and Development, 103 Splaiul Independenței, 050096 Bucharest, Romania; 2 National Institute of Blood Transfusion, 2-8 Dr. C-tin Caracaş, 011155 Bucharest, Romania; 3 Faculty of Medicine, Lucian Blaga University, 2A Lucian Blaga, 550169 Sibiu, Romania; 4 Academic Emergency Hospital, 2-4 Corneliu Coposu Boulevard, 550245 Sibiu, Romania

**Keywords:** Lineage 2 West Nile virus, Eastern European clade, Central/Southern European clade, Co-circulation, Strain replacement

## Abstract

**Background:**

West Nile virus (WNV) is endemic in southeastern Romania and, after the unprecedented urban epidemic in Bucharest in 1996 caused by lineage 1 WNV, cases of West Nile fever have been recorded every year. Furthermore, a new outbreak occurred in 2010, this time produced by a lineage 2 WNV belonging to the Eastern European clade (Volgograd 2007-like strain), which was detected in humans and mosquitoes in the following years.

**Results:**

We report here, for the first time, the emergence, in 2015, of lineage 2 WNV belonging to the monophyletic Central/Southern European group of strains which replaced in 2016, the previously endemized lineage 2 WNV Volgograd 2007-like strain in mosquito populations. The emerged WNV strain harbors H249P (NS3 protein) and I159T (E glycoprotein) substitutions, which have been previously associated in other studies with neurovirulence and efficient vector transmission.

**Conclusions:**

In 2016, both early amplification of the emerged WNV and complete replacement in mosquito populations of the previously endemized WNV occurred in southeastern Romania. These events were associated with a significant outbreak of severe West Nile neuroinvasive disease in humans.

## Background

West Nile virus (WNV) is a mosquito-borne flavivirus and has been circulating in Romania since 1996 when an outbreak with 352 human cases of West Nile neuroinvasive disease (WNND) occurred in southeastern Romania [1]. The outbreak was caused by a WNV strain belonging to genetic lineage 1, and the epidemic vector was identified as *Culex pipiens* [2]. A second significant outbreak (54 cases of WNND in humans), occurred in 2010 and was caused by a WNV of the genetic lineage 2, similar to the strain causing an outbreak in 2007 in Volgograd, Russia [3]. Molecular evidence for the maintenance of this Eastern European WNV for another three transmission seasons (2011–2013) was obtained from mosquito vectors and human patients, suggesting its endemization in southeastern Romania [4]. WNV vertical transmission in *Culex pipiens* was documented for the first time in Europe [4], and the same virus strain was detected in a *Hyalomma marginatum* tick collected from a song thrush [5], suggesting virus maintenance and introduction mechanisms. In 2016, a third significant WNND outbreak in humans was recorded in Romania, with 93 neurological cases [6].

Here, we report, for the first time, detection in Romania, in 2015, of a lineage 2 WNV, belonging to the Central/Southern European monophyletic group of strains [7, 8], co-circulating with previously endemized lineage 2 virus (Volgograd 2007-like strain), and the replacement of the latter by the emerged WNV, in 2016, when a significant outbreak occurred.

## Methods

**Fig. 1:**
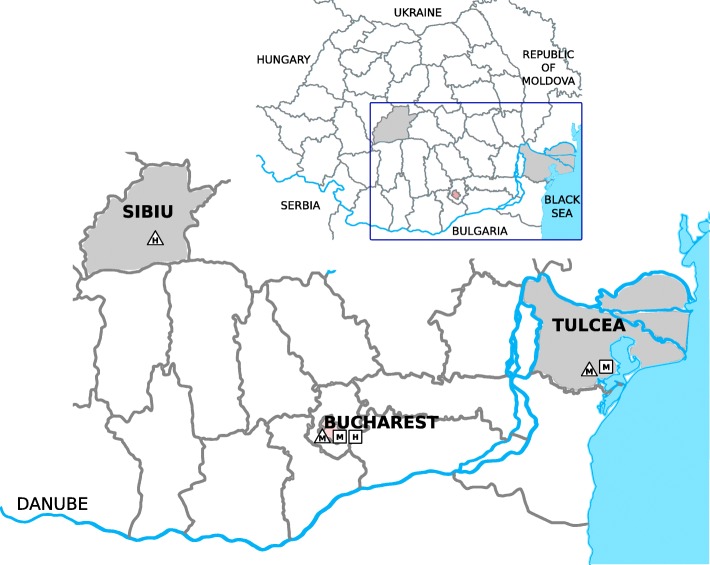
Sample collection sites. *Abbreviations*: M, mosquito sample; H, human sample. Legend: triangle, 2015; square, 2016

Mosquitoes were collected and processed for WNV detection as previously described [9], from May to September 2015–2016, and from two different habitats in southeastern Romania: urban Bucharest, and a natural lagoon habitat, Tulcea county. Two human samples were included in the study and analyzed as previously described [4]: a urine sample, from a WNND case occurring in central Romania, Sibiu county, in 2015, and a plasma sample from an asymptomatic blood donor, detected in 2016, during the outbreak in Bucharest (Table 1 and Fig. 1). Partial NS5 and E glycoprotein sequences were obtained and used for genotyping and phylogeny [4, 10], and partial E and NS3 sequences for identification of non-synonymous substitutions [10, 11]. The other sequences used for inferring phylogeny were retrieved from GenBank.

**Table 1:** West Nile virus lineage 2 strains circulating in Romania, 2015–2016

Study year	Samples	Location	Date of sampling	WNV strain based on NS5 sequence	No. of isolates sequenced	Position 159 in E glycoprotein	Position 249 in NS3 protein
2015	Mosquitoes	Tulcea county site	6-29 August	Eastern European clade	3	M	H
			Aug-28	Central/Southern European clade^a^	1	T	P
		Bucharest city	Jul 9-Aug 19	Eastern European clade	3	na	H
			Aug-20	Central/Southern European clade^a^	1	na	P
	Human	Sibiu	September 11 (onset)	Central/Southern European clade	1	T	P
2016	Mosquitoes	Tulcea county site	Aug 5-Sep 22	Central/Southern European clade^a^	8	T	P
		Bucharest city	Jun-21	Central/Southern European clade^a^	1	na	P
	Human	Bucharest city	Aug-31	Central/Southern European clade^a^	1	T	P

*Abbreviations*: *H* histidine, *M* methionine, *P* proline, *T* threonine, *na* not assessed

^a^Southeastern Romania cluster

## Results

In the transmission seasons 2015–2016, we collected and analyzed 19,390 mosquitoes distributed across 746 pools. Most of them (80%) were captured in Tulcea and only 20% in Bucharest. In Bucharest and Tulcea, respectively, the species composition was: *Culex pipiens* (*s.l.*) (86.97% and 62.52%); *Culex modestus* (7.57% and 3.59%); and *Coquillettidia richiardii* (5.46% and 33.89%).

In 2015, the TaqMan assay used to detect WNV genome was positive in four out of 126 mosquito pools from Bucharest (three *Culex pipiens* and one *Culex modestus* positive pools, 2864 mosquitoes analyzed, minimum infection rate/1000 (MIR) = 1.40), and in 15 out of 352 mosquito pools from Tulcea (ten *Culex pipiens* and five *Coquillettidia richiardii* positive pools, 9385 mosquitoes analyzed, MIR = 1.60). In 2016, nine out of 41 mosquito pools were positive in Bucharest (seven *Culex pipiens* and two *Culex modestus* positive pools, 1072 mosquitoes analyzed, MIR = 8.40), and 25 out of 227 in Tulcea (25 *Culex pipiens* positive pools, 6069 mosquitoes analyzed, MIR = 4.12). Seventeen out of these positive mosquito pools yielded amplification products for partial sequences of NS5, twelve pools for NS3, and three pools for E region (Table 1). In 2015, WNV infected mosquitoes were first detected on July 9 and 17 in Bucharest and Tulcea, respectively, while in 2016 the first infected mosquitoes were detected on June 21 in Bucharest, and June 6 in Tulcea, revealing early virus amplification, which led to higher mosquito infection rates in the outbreak year.

**Fig. 2:**
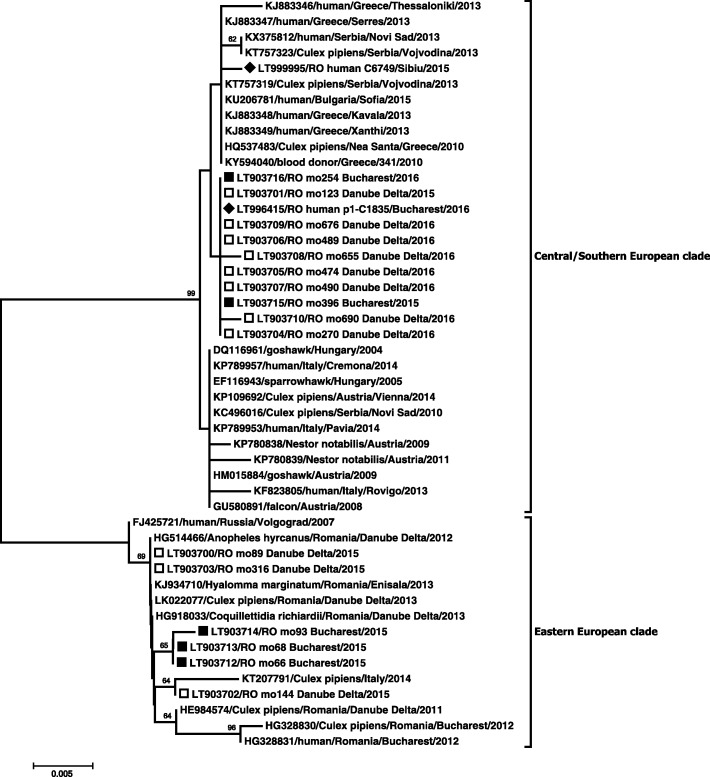
Phylogenetic tree for lineage 2 West Nile virus strains circulating in Romania, 2015–2016. Black diamond: sequence obtained in this study from human samples; black square: sequence obtained in this study from mosquito samples collected in Bucharest; white square: sequence obtained in this study from mosquito samples collected in Tulcea county. The other sequences included in the analysis were retrieved from GenBank. Numbers at nodes represent bootstrap percentages (values < 50% are not shown). Phylogenetic relatedness was inferred with from a 593 nt sequence spanning NS5 region (nt positions 9442–10,034 in isolate Nea Santa-Greece-2010, GenBank: HQ537483) using the Neighbor-Joining method, Kimura 2-parameter and 1000 bootstrap replicates

In 2015, in the urine sample of a WNND patient, a resident of Sibiu county, central Romania, a WNV belonging to the Central/Southern European clade of genetic lineage 2 was detected. The strain was highly similar to the group of strains having the Nea Santa-Greece-2010 WNV lineage 2 strain as its prototype which has been circulating in the Balkans since 2010 (Table 1, Figs. 1 and 2).

In southeastern Romania, in 2015, the endemically established Volgograd 2007-like strain was found co-circulating in mosquito populations with a WNV strain, also closely related to the Nea Santa 2010 group of strains. However, in 2016, only this newly emerged WNV strain was detected in mosquitoes in the study sites, and a very similar isolate was detected in a blood donor sample collected during the outbreak peak in Bucharest (Table 1, Figs. 1 and 2). All the obtained NS3 sequences of these newly detected isolates displayed the H249P substitution, as seen in the Nea Santa strain, but the E fragment differed from the latter by I159T substitution (Table 1). Despite the limited resolution of NS5 fragment used for inferring phylogeny, the analysis showed that all the isolates of the newly detected virus in southeastern Romania form a cluster, which does not include the WNV derived from the patient in Sibiu, central Romania (Table 1, Fig. 2).

## Discussion

Lineage 2 WNV was first detected in Romania as Eastern European clade [5] and caused the human outbreak of WNND in 2010 [3]. Molecular evidence for its maintenance between 2011–2015, in mosquitoes and humans, in southeastern Romania, was provided by Dinu et al. [4] and the present study. Our results showed that in this region, the emerged WNV strain belonging to the Central/Southern European genetic lineage 2 was first found in 2015 in mosquitoes, co-circulating with the Volgograd 2007-like strain, while in 2016 it completely replaced the latter in mosquito populations. The emerged WNV strain was also detected in a human sample collected in Bucharest at the peak of the outbreak, suggesting its involvement in the 2016 epidemic. The records of the European Centre for Disease Prevention and Control [6] showed that between July 1 - October 10, 2016, 93 human WNND cases were reported in Romania, most of them (68 cases) distributed in the southeast of Romania, including Bucharest city (18 cases); re-emergence of severe WNND cases with high mortality was also observed in this area [12]. Only four WNND cases were recorded beyond the Carpathian Mountains, in Transylvania, in 2016 [6], where a WNV of the Central/Southern European lineage 2 was detected in 2015 and described in the present study. However, this isolate is distinct from the isolates of 2015–2016 southeastern Romania WNV cluster. Co-circulation of different strains of the Central/Southern European clade was also reported in Serbia [8]. Multiple introduction events and local evolution may lead to a high diversity of WNV, as described in Italy, where co-circulation of distinct lineages and different clades of lineage 2 was documented [8, 13].

Molecular evolution with impact on viral fitness and neurovirulence in lineage 2 WNV strains mirrors that of lineage 1 [14]. In both lineage 2 strains circulating in Romania, the E glycoprotein presents an N-linked glycosylation site (position 154), which was previously related to neuroinvasiveness [15], and efficient virus transmission in vectors [16]. In the strains emerged in 2015–2016, in southeastern and central Romania, at amino acid position 159 (E glycoprotein), isoleucine was substituted by threonine. In lineage 1, V159A substitution was related to the emergence of the North American genotype WN02 which displaced the introduced NY 1999 strain and was linked to efficient vector transmission and dissemination [17]. Threonine in this position was found in an Italian lineage 2 cluster and was predicted to be subject of positive selection [8]. H249P substitution in NS3 was associated with neurovirulence and increased and sustained viremia in birds, leading to high epizootic potential [18].

## Conclusions

Lineage 2 WNV strains belonging to the monophyletic Central/Southern European group of strains and harboring H249P (NS3 protein), and I159T (E glycoprotein) substitutions, were for the first time detected in Romania in 2015. The emerged WNV in southeastern Romania formed a distinct cluster from the isolate detected in central Romania in 2015. In 2016, early amplification of the emerged WNV, and complete replacement in mosquito populations of the previously endemized virus in southeastern Romania occurred. These events were associated with a human outbreak of severe, high fatality WNND. Although limited, the data obtained in this study contribute to the whole picture of the adaptive evolution in Europe of the WNV clade derived from the Hungarian prototype strain.

## Abbreviations

E: Envelope glycoprotein
MIR: Minimum infection rate/1000
NS3: Nonstructural protein 3
NS5: Nonstructural protein 5
WNND: West Nile neuroinvasive disease
WNV: West Nile virus
